# Effects of HMW-GSs on quality related traits in wheat (*Triticum aestivum* L.) under different water regimes

**DOI:** 10.1371/journal.pone.0237711

**Published:** 2020-08-18

**Authors:** Jiajia Zhao, Xingwei Zheng, Ling Qiao, Chuan Ge, Bangbang Wu, Shuwei Zhang, Linyi Qiao, Zhiwei Feng, Jun Zheng

**Affiliations:** 1 Institute of Wheat Research, Shanxi Agricultural University, Linfen, Shanxi, China; 2 Institute of Science and Technology Information of Shanxi, Taiyuan, Shanxi, China; 3 Academy of Organic Dry Farming Agricultural Research, Shanxi Agriculture University, Taiyuan, Shanxi, China; North Dakota State University, UNITED STATES

## Abstract

Alleles at the *Glu-1* loci play important roles in the functional properties of wheat flour. The effects of various high-molecular-weight glutenin subunit (HMW-GS) compositions on quality traits and bread-making properties were evaluated using 235 doubled haploid lines (DHs). The experiment was conducted in a split plot design with two water regimes as the main plot treatment, and DH lines as the subplot treatments. Results showed that the presence of subunit pair 5+10 at the *Glu-D1* locus, either alone or in combination with others, appears to provide an improvement in quality and bread-making properties. At the *Glu-A1* locus, subunit 1 produced a higher Zeleny sedimentation value (Zel) and stretch area (SA) than subunit 2* when subunits 14+15 and 5+10 were expressed at the *Glu-B1* and *Glu-D1* loci, and 2* had a positive effect on the maximum dough resistance (Rmax) when subunits 14+15 and 5'+12 were expressed at the *Glu-B1* and *Glu-D1* loci, respectively. Given subunit 1 at the *Glu-A1* locus and 5'+12 at the *Glu-D1* locus, the effects of *Glu-B1* subunits 14+15 on the tractility (Tra), dough stability time (ST), and dough development time (DT) under the well-watered regime were significantly higher than those of *Glu-B1* subunits 13+16. However, 13+16 had a positive effect on SA under the rain-fed regime when subunits 2* and 5+10 were expressed at the *Glu-A1* and *Glu-D1* loci, respectively. Multiple comparisons analysis revealed that the Zel and Rmax of the six subunits and eight HMW-GS compositions were stable under different water regimes. Overall, subunit compositions 1, 13+16 and 5+10 and 1, 14+15 and 5+10 had higher values for quality traits and bread-baking properties under the two water regimes. These results could play a positive guiding role in selecting and popularizing varieties suitable for production and cultivation in local areas.

## Introduction

Grain protein content (GPC) plays an important role in end-use quality and thus determines the economic value of the crop. Improvement in GPC is a major objective in wheat breeding programs around the world [1]. Grain proteins are generally classified into four groups, albumins, globulins, prolamins, and glutelins [1]. Among the three major food crops (rice, wheat, and maize), wheat (*Triticum aestivum* L.) is unique in accumulating gluten proteins in its grains. Gluten proteins contain roughly equal amounts of gliadins and glutenins, which are important factors influencing elasticity, extensibility, and viscosity of dough [2, 3]. Glutenins are composed of high- and low-molecular-weight glutenin subunits (HMW-GSs and LMW-GSs, respectively), which form gluten macropolymers stabilized by inter-chain disulfide and hydrogen bonds [4, 5]. Although HMW-GS only account for 10% of total proteins, they explain approximately 60% of the phenotypic variation in bread-making quality [6, 7]. HMW-GSs are encoded by multiple alleles, and the glutenin content and ration can be influenced by environmental factors [8].

In the past few decades, more than 100 allelic variations in HMW-GSs have been reported in bread wheat [9], which play important roles in dough elasticity, dough strength, and bread-making properties [8, 10–13]. In general, the *Glu-D1* locus has the strongest effect, followed by the *Glu-B1* and *Glu-A1* loci [14, 15]. Subunits at *Glu-1* loci have different effects on quality parameters. Previous studies on the relationship between glutenin subunits and flour quality have established inconsistent results [6, 16–21]. Grama et al. [18] demonstrated that contributions to Zeleny sedimentation value (Zel) and bread volume rank as 7+8 > 17+18 = 13+16 > 7+9, while rankings of 17+18 = 13+16 = 7+8 > 7+9 > 6+8 > 7 and 14+15 > 17+18 > 7+8 > 6+8 > 7 have also been reported [19, 20]. At the *Glu-D1* locus, the contribution of 5+10 to dough quality is significant over other alleles [17], while Pena et al. [21] reported that the contribution of 5'+12 on Zel, strength, extensibility, and bread volume was higher than that of 5+10. It is difficult to conclusively evaluate the contribution ranking of *Glu-1* alleles, since most materials used in previous studies have different genetic backgrounds. Recombinant inbred lines (RILs), near isogenic lines (NILs), and doubled haploid lines (DHs) are ideal materials for accurately reflecting the effects of HMW-GSs on quality, as well as eliminating background influences, such as LMW-GSs, gliadin, starch, and lipids [22, 23]. Based on NILs, Gao et al. [24] demonstrated that the effect of subunits 17+18 and 14+15 on dough stability time were higher than those of 7+8 and 7+9. Using RILs, a previous study showed that subunits 7+8 had a more positive effect on dough extensibility than 17+18 [25]. Subunits 5+10 in DHs had a more positive contribution to dough properties than 2+12 [26]. However, the differences and interactions of other known quality related subunits, particularly some favorable subunits such as 1, 2*, 13+16, and 14+15, have not been evaluated using RILs, NILs and DHs, mainly due to a lack of suitable and available materials.

It is noteworthy that HMW-GSs are genetically controlled and stable in terms of heredity. In contrast, the expressions or amounts of HMW-GSs and the proportion of each subunit to the total subunits amount can be modified by environmental conditions, such as drought, waterlogging, mineral nutrition, and temperature [27–30]. Water deficit affects the expression and accumulation of the grain proteome and causes significant changes to the composition of storage proteins and gluten quality [28, 31]. Compared with proteins, gluten polymers (GMP) are more sensitive to the environment. HMW-GSs are believed to be major contributors to glutenin particles in GMP [32] and could explain 44% of the total variation in gluten quality [33]. The concentrations of HMW-GSs and GMP in the grain were up-regulated by an early drought [28]. The GMP particle size of subunits 5+10 and 2+12 at the *Glu-D1* locus and 14+15 at the *Glu-B1* locus were not affected by water deficit, while subunits 1 and 7+8 were significantly affected [34]. Comparative proteomics analysis also revealed that water deficiency resulted in significant upregulation of 12 HMW-GSs, including subunits 7+9 and 2+12 [35].

Comprehensive studies on the association and quantitative effects between favorable subunits and quality related traits under different water regimes are still limited. In this study, DHs, which consisted of different favorable subunits at *Glu-1* loci, were evaluated with various quality traits to understand the effect of HMW-GSs on dough quality and bread-making properties under different water regimes. The results of this study could benefit wheat breeding programs concerning grain quality of different ecological types.

## Materials and methods

### Plant materials

In total, 250 lines of a DH population derived from a cross between Jinchun 7 and L1219 were analyzed in terms of the allelic composition of the *Glu-1* loci. Jinchun 7 (awnless 1 × Sharek) was approved by Shanxi Variety Committee in 1982, and has subunits 1, 13+16, and 5+10 at the *Glu-1* loci. L1219 is a strain selected by our laboratory, which has subunits 2*, 14+15, and 5'+12 at the *Glu-1* loci. Field trials were performed from 2016–2018 in Linfen, Shanxi province, China, located at 36°2' N and 111°18' E. The experiment was conducted in a split plot design with two water regimes as the main plot treatment, and DHs as the subplot treatment in randomized complete blocks with two replicates. Every plot area in the experiment was 6 m^2^. Two water regimes design as rain-fed (RF) and well-watered (WW) treatments. At the wintering, jointing and filling stages, the well-watered treatment group was irrigated with 700 m^3^/hm^2^, while the rain-fed group had no irrigation. The average rainfall was 222.7 mm during the growing season over the two-years study periods (S1 Fig). Field managements was in accordance with local practice and no pesticides were used. Normal mature grains were harvested for the grain and flour characteristics test.

### Identification of HMW-GSs

Glutenin proteins were extracted according to Singh et al. [36] and prepared for SDS-PAGE analysis. HMW-GSs were classified following the nomenclature of Payne and Lawrence [8]. The standards were Chinese Spring (N, 7+ 8, 2+ 12), Shiluan02-1 (1, 7+9, 5+10), Shaan225 (1, 14+15, 2+12), and Yannong19 (1, 17+18, 5+10). HMW-GS composition was initially determined by SDS-PAGE, but the SDS-PAGE of alleles at the *Glu-A1* locus were difficult to distinguish. Subsequently, we verified allele by the polymerase chain reaction (PCR) using allele-specific markers for the detection of alleles (1 and 2*) at the *Glu-A1* locus (S1 Table), as described by Liu et al. [37].

### Measurement of quality traits

Lines with the same subunit combination were mixed in equal amounts for standard farinograph and extensograph analyses. The protein content (14% MB) was recorded using near infrared analysis (Perten, Sweden). Grain samples were adjusted to 14% moisture according to grain hardness and milled on a Brabender Quadrumat Junior laboratory mill (Brabender, Duisburg, Germany), as described by Salmanowicz et al. [38]. According to American Association of Cereal Chemists (AACC) Method 56–63, weighed 0.32 g into a 10 mL stoppered graduated cylinder, then simultaneously started timing and added 5 mL bromphenol blue solution to the graduated cylinder. Thoroughly mixed flour and water, and all flour was swept completely into suspension. Then place the cylinder in a mixer and mixed for 5 min. After that, added 5 mL lactic acid reagent, returned the cylinder to the mixer, and mixed until 10 min had elapsed from start of timing. Following this, removed the cylinder from the mixer, immediately placed it in an upright position, and read the volume of sediment after standing for 5 min, which constituted the Zeleny sedimentation value. The rheological properties of the dough, including dough development time, stability time tractility, stretch area, and maximum resistance (Rmax), were evaluated using the Brabender Farinograph (AACC-54-21) and Brabender Extensograph (AACC-54-10). Each sample was replicated three times.

### Bread making and quality evaluation

Lines with the same subunit combination lines were mixed in equal amounts for bread-making. Bread-making was performed according to the China national standard *Bread Baking Quality of Wheat Flour—Straight Dough Method* (GB/T35869-2018). The ingredients (100 g flour, 6.0 g sucrose, 1.5 g salt, 3.0 g shortening, 2.7 g yeast, 40 ppm ascorbic acid solution, and an adequate volume of distilled water) were mixed in a 100 g Micro-Mixer. Dough was punched and fermented at 38°C and 85% RH for 60 min with a second punching at 20 min, then molded and placed in pup loaf baking pans (inner diameter of upper mouth 14.3 cm × 7.9 cm, bottom inner diameter 12.9 cm × 6.4 cm, depth 5.7 cm). The molded dough was baked in an oven for 20 min at 215°C. After cooling, bread volume was measured using a BVM6640 (Perten, Sweden), after which the bread was placed in a constant temperature and humidity storage box. After 18 h, the bread score was evaluated according to GB/T35869-2018, in which a full score was 100, including bread volume (45), external appearance (5), color (5), texture (10), and structure (35). All parameters are subjective measurements, except for bread volume.

### Statistical analysis of data

The correlation coefficients of traits and analysis of variance were determined using SAS statistical software (SAS Institute Inc., Cary, NC, USA). Least significant difference (LSD) multiple comparisons were carried out to examine the effects of individual subunit, HMW compositions, and water regimes on quality. Percentage ranges were calculated to determine the effects of a change in water regimes on the value of each quality parameter, using the formula: 100[(maximum value) − (minimum value)] / mean value [25].

## Results

### HMW-GS genotype characterization

SDS-PAGE and PCR methods consistently showed that the parents of the DHs (Jinchun 7 and L1219) differed in HMW-GS composition. Jinchun 7 has subunits 1, 13+16, and 5+10 at the *Glu-1* loci, while L1219 has subunits 2*, 14+15, and 5'+12. The HMW-GS compositions of all 250 DH lines were determined, and the lines with renewed HMW-GS composition were eliminated, leaving 235 lines for further analysis. In total, eight HMW-GS combinations were distinguished from the DH lines, which constituted 8 groups of lines with different allelic variants at*Glu-1* loci (Table 1, S2 Fig). The two parental types, SC2 and SC7, were found at frequencies of 13.6% and 10.6%, respectively. And the frequencies of occurrence of the other six recombinant types, SC1, SC3, SC4, SC5, SC6, and SC8, were 8.9%, 14.5%, 10.6%, 10.6%, 17.4% and 13.6%, respectively.

**Table 1:** Occurrence of HMW-GSs in the DHs.

*Code*	*Glu-A1*	*Glu-B1*	*Glu-D1*	Number of DHs	Ratio (%)
**SC 1**	1	13+16	5'+12	21	8.9
**SC 2**	1	13+16	5+10	32	13.6
**SC 3**	1	14+15	5'+12	34	14.5
**SC 4**	1	14+15	5+10	25	10.6
**SC 5**	2*	13+16	5'+12	25	10.6
**SC 6**	2*	13+16	5+10	41	17.4
**SC 7**	2*	14+15	5'+12	25	10.6
**SC 8**	2*	14+15	5+10	32	13.6

SC indicates subunit composition.

### Performance of quality related traits in DHs

The correlation between quality related traits over the two-years study periods was highly significant (S2 Table) and the average value was used for further analysis. However, the quality traits were less significantly correlated between the two water regimes (S2 Table). Quality analysis showed that quality traits under the rain-fed regime were better than those under the well-watered regime (Table 2). There were significant differences in Protein content (PC), Tractility (Tra), dough stability time (ST), and stretch area (SA) between two water regimes, while no significant differences were found between two water regimes in Zeleny sedimentation value (Zel), maximum resistance (Rmax) and development time (DT) (S3 Table).

**Table 2:** Variation in quality traits of DHs in different water regimes.

Treatment	Traits	Parents		DHs		
		Jinchun 7	L1219	Mean	Range	CV (%)
**WW**	**PC (%)**	15.31 ± 0.91a	14.82 ± 0.28a	14.98	14.67–15.28	1.32
	**Zel (mL)**	39.76 ± 1.81a	35.96 ± 4.94a	36.55	35.05–40.01	5.12
	**Tra (mm)**	164.42 ± 2.88a	159.51 ± 1.13a	162.15	156.81–165.29	1.85
	**Rmax (B.U)**	540.24 ± 12.19a	520.33 ± 33.12a	523.99	485.56–547.21	3.95
	**ST (min)**	6.94 ± 0.30a	6.73 ± 0.63a	6.79	6.33–6.92	3.17
	**DT (min)**	5.40 ± 0.21a	5.17 ± 0.77a	5.23	4.98–5.45	3.46
	**SA (cm**^**2**^**)**	98.97 ± 4.98a	95.08 ± 2.24 a	96.28	90.09–100.95	3.36
**RF**	**PC (%)**	16.43 ± 0.02a	16.07 ± 0.10a	16.22	15.93–16.50	2.00
	**Zel (mL)**	39.21 ± 3.87a	37.56 ± 2.22a	37.39	35.92–39.43	3.61
	**Tra (mm)**	178.68 ± 5.72a	171.74 ± 6.35a	173.59	170.65–177.74	1.38
	**Rmax (B.U)**	558.99 ± 16.64a	515.36 ± 12.99a	528.52	500.94–563.925	3.74
	**ST (min)**	8.07 ± 1.01a	7.86 ± 0.11a	7.57	7.16–8.01	3.75
	**DT (min)**	5.39 ± 0.17a	5.28 ± 0.04a	5.26	5.13–5.36	1.62
	**SA (cm**^**2**^**)**	107.09 ± 0.53a	98.59 ± 3.82a	105.31	97.10–113.34	5.57

RF = rain-fed regime; WW = well-watered regime.

Parental cultivars Jinchun 7 and L1219 differed only slightly in terms of quality traits. Means of these parameters in DH lines were approximately mid-parent values. Values of quality traits in the studied DH lines fell within a wide range (Table 2). PC had the smallest variation among all the assessed traits. Compared with PC, Zel, Rmax, ST, and SA showed wider variation ranges and larger coefficients of variation. The variation for quality traits in DHs, especially the Zel (WW) and SA (RF) were influenced by HMW-GS compositions.

### Effects of subunit on quality traits

In order to understand the effects of glutenin subunits on quality related traits, LSD multiple comparisons were performed. Generally, lines with subunits 1 and 2* were characterized by better dough quality. The contribution of subunit 1 was significantly higher (3.48%) than that of subunit 2* for Zel(WW). The other quality traits at the *Glu-A1* locus were ranked as 1 > 2* with no significant difference, whereas Rmax(WW) and SA(WW) of lines with subunit 2* were higher than that of subunit 1. No significant difference was found in quality traits for the studied DH lines differing in *Glu-B1* subunits in both water regimes. The effects of subunits 5+10 at the *Glu-D1* locus were significantly better than those of 5'+12 for Zel, Rmax, and DT(WW). Overall, the quality traits at the *Glu-A1* and *Glu-D1* loci were ranked as 1 > 2* and 5+10 > 5'+12, respectively. The highest Zel value of the *Glu-B1* locus was detected for 13+16 and the values of other quality traits were ranked as 14+15 > 13+16.

### Effects of HMW-GS compositions on quality traits

Similar protein contents were observed from the two water regimes among eight HMW-GS compositions, ranging from 14.82% to 15.28% and 15.93% to 16.50% in the WW and RF regimes, respectively (Table 3). However, effects on other quality traits were varied among the eight HMW-GS compositions. Mean values of quality parameters in DH lines differing in subunit composition at the *Glu-1* loci are shown in Table 3.

**Table 3:** Effects of glutenin subunits on quality traits.

	PC (%)		Zel (mL)		Tra (mm)		Rmax (B.U)		ST (min)		DT (min)		SA (cm^2^)	
	WW	RF	WW	RF	WW	RF	WW	RF	WW	RF	WW	RF	WW	RF
**SC1**	14.83a	16.33a	35.05c	36.10b	156.81b	170.65b	504.24bc	500.94c	6.33b	7.16b	5.01c	5.13a	90.09b	98.67b
**SC2**	15.22a	16.50a	40.01a	39.43a	163.05ab	175.54ab	538.87a	539.62ab	6.91a	7.74a	5.38ab	5.30a	98.09a	106.42ab
**SC3**	14.99a	16.00a	35.11bc	35.92b	165.06a	177.74a	485.56c	522.85bc	6.89a	7.89a	5.18abc	5.36a	97.46a	111.06a
**SC4**	15.28a	16.27a	38.52a	38.87a	165.29a	173.84ab	547.21a	537.02ab	6.92a	8.01a	5.38ab	5.32a	98.25a	113.34a
**SC5**	14.67a	15.93a	35.32bc	36.17b	158.61ab	171.25ab	519.54ab	507.42c	6.58ab	7.45ab	4.98c	5.13a	94.43ab	103.22ab
**SC6**	14.90a	16.04a	37.30ab	38.35a	163.54ab	174.97ab	522.24ab	563.92a	6.91a	7.50ab	5.45a	5.25a	100.95a	109.70a
**SC7**	14.98a	16.28a	35.84bc	37.09ab	162.91ab	172.45ab	518.69ab	523.14bc	6.88a	7.37ab	5.11bc	5.30a	95.65ab	102.90ab
**SC8**	15.00a	16.45a	35.21bc	37.18ab	161.91ab	172.28ab	535.60a	533.28ab	6.86a	7.45ab	5.32ab	5.26a	95.33ab	97.10b

Values followed by different letters and significantly different at 5% probability level.

#### Under well-watered regime

The highest Zel (40.01 mL) was detected in SC2, followed by SC4. These values were significantly higher than those of other subunit compositions except SC6. For subunits 14+15 at *Glu-B1* and 5+10 at *Glu-D1*, DH lines with subunit 1 showed significantly higher Zel than those for lines with 2*. SC2 also showed significantly higher Rmax, ST, DT, and SA than SC1 (Table 3). For Tra and ST, the contribution of SC3 were greater than those of SC1 at the 5% probability level, indicating that subunits 14+15 at the *Glu-B1* locus can improve Tra and ST by 5.26% and 8.85%, respectively, compared with 13+16. Additionally, the ST values of SC2, SC4, SC6, SC7, and SC8 were significantly higher than that of SC1.There was a more significant effect on Rmax for subunits 5+10 at the *Glu-D1* locus than that for 5'+12. For example, the Rmax values of SC4 and SC2 were significantly higher than those of SC1 and SC3. Additionally, the effect of subunit 2* was more prominent than that of subunit 1 for Rmax when the subunits 14+15 and 5'+12 were expressed at the *Glu-B1* and *Glu-D1* loci, respectively.

However, the highest DT and SA values were detected in SC6, followed by SC2 and SC4, while SC1 was the lowest. The subunits at the *Glu-D1* locus had significantly different effects on DT for subunit 1 or 2* at *Glu-A1* and 13+16 at *Glu-B1*. For example, the effects in SC2 and SC6 were obviously greater than in SC1 and SC5. Conversely, the effect of 5+10 on SA was higher than that of 5'+12 when subunits 1 and 13+16 were expressed at *Glu-A1*and *Glu-B1* loci, respectively.

#### Under rain-fed regime

Compared to the well-water regime, there were no significant differences in DT among the eight HMW-GS compositions. For Zel, the values detected in SC2, SC4, and SC6 were significantly higher than those of SC1, SC3, and SC5. The Rmax value of SC2 was higher than that of SC1 by 7.72%, and the Rmax value of SC6 was 11.13% higher than that of SC5. These results indicated that subunits 5+10 have more positive effects on Zel and Rmax than 5'+12 under the rain-fed regime.

Similar to the WW regime, subunits at the *Glu-B1* locus showed a significant difference concerning Tra and ST for subunits 1 at *Glu-A1* and 5'+12 at *Glu-D1*. For example, SC3 was higher than SC1 at the 0.05 probability level. The highest Tra was detected in SC3 and the highest ST was detected in SC4.

SA values of SC4, SC2, and SC6 were significantly higher than those for SC1 and SC8, indicating that the contribution of subunit 1 was greater than that of subunit 2* with subunits 14+15 at *Glu-B1* and 5+10 at *Glu-D1*. Additionally, the effects of subunits at the *Glu-B1* locus differed greatly depending on the presence of subunits at the *Glu-A1* and *Glu-D1* loci. Given that subunit 2* expressed at the *Glu-A1* locus and 5+10 expressed at the *Glu-D1* locus, the contribution of 13+16 was greater than that of 14+15, while it was opposite for the subunit 1 at *Glu-A1* and 5'+12 at *Glu-D1*. These results indicated that subunits of different HMW-GS compositions perform differently concerning the quality traits.

#### Interaction between allelic combinations

Effects of the interactions among subunits at the *Glu-1* loci on quality traits were varied. For subunit 1 at the *Glu-A1* locus, lines with subunits 13+16 and 5+10 had higher Zel and Rmax (WW) than those with subunits 14+15 and 5'+12. In addition, the interaction of 14+15 and 5+10 showed that the effect on Zel, Rmax (WW), ST, DT (WW), and SA were greater than that of 13+16 and 5'+12 according to the performance of HMW-GS composition SC1 (1, 13+16, 5'+12) and SC4 (1, 14+15, 5+10). More significant effects on Zel and DT (WW) were found for interactions of 1 and 5+10, and 2* and 5+10 than those of 2* and 5'+12, and 1 and 5'+12, respectively. In short, the presence of 5+10 at *Glu-D1*, either alone or in combination with any other subunits at the *Glu-A1* and *Glu-B1* loci, appeared to compromise quality improvement.

### Influence of different water regimes on quality

Six subunits showed significant differences between the two water regimes in their effects on PC, Tra, ST, and SA, while the effects on Zel and Rmax showed no significant differences (S4 Table). For DT under the RF regime, the effect of subunits 5'+12 was significantly higher than that under the WW regime (Table 4 and S4 Table). Allelic variation at the *GIu-A1* and *GIu-B1* loci had no significant differences for DT between the two water regimes. Percentage ranges for quality traits of subunits 5'+12 were higher than those of 5+10, indicating that quality traits of lines with subunits 5'+12 tend to be influenced by water condition (S4 Table).

**Table 4:** Comparison of the effects produced by alleles at the *Glu-1* loci on quality traits.

Loci	subunits	PC (%)		Zel (mL)		Tra (mm)		Rmax (B.U)		ST (min)		DT (min)		SA (cm^2^)	
		WW	RF	WW	RF	WW	RF	WW	RF	WW	RF	WW	RF	WW	RF
***Glu-A1***	2*	14.89a	16.18a	35.92b	37.20a	161.74a	172.74a	524.02a	531.94a	6.71a	7.44a	5.22a	5.24a	96.59a	103.23a
	**1**	15.08a	16.28a	37.17a	37.58a	162.55a	177.69a	518.97a	525.11a	6.76a	7.70a	5.23a	5.28a	95.97a	107.37a
***Glu-B1***	**14+15**	15.06a	16.25a	36.17a	37.27a	165.18a	173.84a	521.77a	529.07a	6.89a	7.68a	5.25a	5.31a	96.67a	106.10a
	**13+16**	14.91a	16.20a	36.92a	37.51a	163.79a	172.86a	521.22a	527.98a	6.68a	7.46a	5.21a	5.21a	95.89a	104.50a
***Glu-D1***	**5+10**	15.10a	16.31a	37.76a	38.46a	163.45a	174.16a	535.98a	543.46a	6.90a	7.68a	5.38a	5.28a	98.16a	106.64a
	**5'+12**	14.86a	16.13a	35.33b	36.32b	160.85a	173.02a	507.01b	513.59b	6.67a	7.47a	5.07b	5.23a	94.41a	103.96a

Values followed by different letters and significantly different at 5% probability level.

Effects of the eight HMW subunit compositions on quality traits between the two water regimes are shown in Table 5. PC and Tra of the eight groups (SC1-SC8) were influenced by water conditions, with SC1 being the most affected. Zel, Rmax and DT of all groups displayed no significant differences between WW and RF, and these traits are mainly controlled by genotypes and thus were barely affected by water factors. Although ST and SA values of all individual subunits at the *Glu-1* loci were significantly affected by water conditions, ST of SC7 and SC8 showed no significant differences between the two water regimes, and for SA, only SC3 and SC4 were significantly influenced. This indicated that the interactions among subunits at the *Glu-1* loci influenced the quality of wheat.

**Table 5:** Effects of HMW-GS on quality parameters between different water regimes.

	PC (%)		Zel (mL)		Tra (mm)		Rmax (B.U)		ST (min)		DT (min)		SA (cm^2^)	
	F	%	F	%	F	%	F	%	F	%	F	%	F	%
**SC1**	19.09**	9.63	0.46	2.95	11.90**	8.45	0.01	0.66	9.28**	12.31	1.92	2.37	2.38	9.09
**SC2**	10.87**	8.07	0.09	1.46	8.57**	7.38	0.00	0.14	7.50**	11.33	0.17	1.50	1.32	8.15
**SC3**	14.81**	6.52	0.05	2.28	5.82**	7.40	3.24	7.40	7.14**	13.53	0.69	3.42	4.16*	13.04
**SC4**	18.84**	6.68	0.04	0.90	6.07**	5.04	0.14	1.88	13.50**	14.60	0.15	1.12	6.64*	14.26
**SC5**	21.93**	8.24	0.30	2.38	12.89**	7.66	0.19	2.36	14.24**	12.40	1.49	2.97	2.71	8.89
**SC6**	17.22**	7.37	0.46	2.78	18.75**	6.75	3.18	7.67	4.62*	8.19	0.59	3.74	3.81	8.31
**SC7**	21.90**	8.32	0.45	3.43	8.40**	5.69	0.18	0.85	2.55	6.88	2.05	3.65	1.71	7.30
**SC8**	10.33**	9.22	0.90	5.44	6.11**	6.21	2.10	0.43	3.34	8.25	0.20	1.13	0.08	1.84

F is the test value of the analysis of variance between rain-fed and well-watered regimes.

* and ** represent significant differences at the 0.05 and 0.01 levels, respectively. % represents percentage ranges to determine the effects of a change in the two water regimes on the value of each quality parameter.

### Effect of HMW-GS on bread-making quality

**Fig 1:**
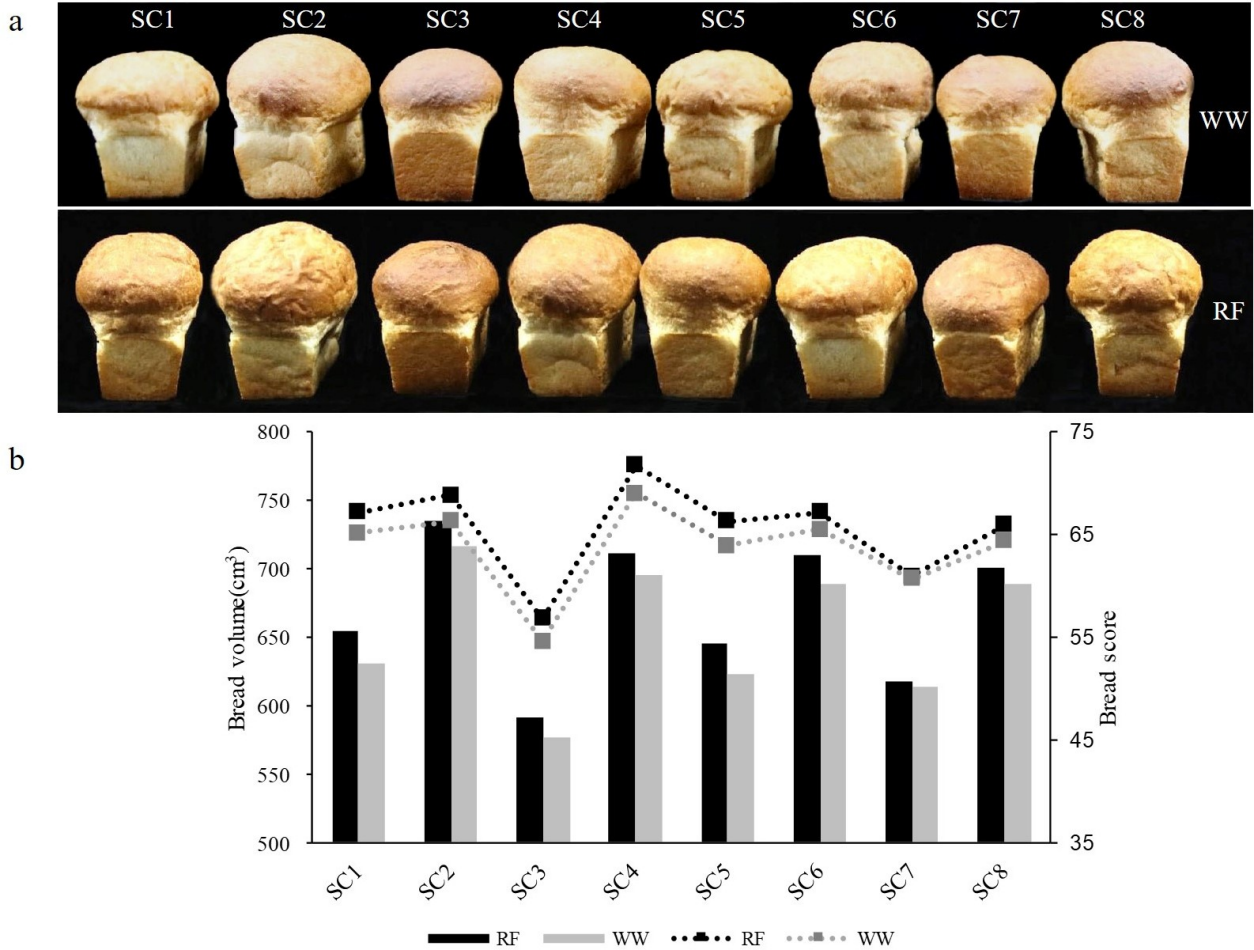
Effects of HMW-GS on bread-making properties under two water regimes. (a) Bread with different subunit combinations under two water regimes, (b) bread volumes and scores of different HMW-GS combinations under two water regimes.

Generally, allelic variation in *Glu-1* had a greater effect on bread-making quality. Payne et al. [17] reported that up to 67% of genetic variation for bread-making qualities was conditioned by the HMW-GS composition. Values of bread baking parameters in the eight groups (SC1-SC8) are shown in Fig 1 and S5 Table. Large differences among the groups were observed for bread parameters.

SC1, SC2 and SC4, which all contained subunit 1, showed higher bread volumes and bread scores than SC5, SC6 and SC8, which all contained subunit2*. Conversely, comparison between SC3 and SC7 showed that bread volume and bread score of subunit 2* were greater than those of subunit 1 in the composition of 14+15 and 5'+12 (Fig 1B). This indicated that the relationship of subunits interaction to bread-quality was varied. SC1, SC2, SC5, and SC6, which all contained subunits 13+16 at *Glu-B1* locus, showed higher bread-making parameters than SC3, SC4, SC7, and SC8, which all contained subunits 14+15 (Fig 1B). Comparison of bread-making parameters among groups with different *Glu-D1* subunits (Fig 1B) showed that SC2, SC4, SC6 and SC8 with 5+10 were significantly higher than SC1, SC3, SC5 and SC7 with 5'+12. These results are in accordance with a previous study which showed that subunits 5+10 had superior dough quality [17]. SC2 and SC4 were characterized by the highest bread-making quality compared to the other groups. This indicated that subunits 5+10 had a more positive effect on bread properties when subunit 1 was expressed at *Glu-A1* locus and subunits 13+16 or 14+15 were expressed at *Glu-B1* locus. The highest bread volume was detected in SC2, while the highest bread score was detected in SC4, which had a better bread crumb structure (S5 Table).

Generally, the protein content, gluten quality, and bread-making quality were increased in wheat under water-deficit [39]. The present results are consistent with the previous study reporting better baking quality under RF regime than under WW (Fig 1B). Different water regimes resulted in slight changes in bread scores for SC8 and SC7 (Fig 1B, S5 Table), which was associated with the lack of significant differences concerning the ST of SC7 and SC8 between RF and WW.

## Discussion

### Ideal materials for evaluating effects of HMW-GSs on quality

The effects of glutenin subunits on quality of wheat have been previously reported [17–20]. NILs, RILs, and DHs carrying different alleles of glutenin developed in a similar genetic background are ideal materials to evaluate HMW-GSs’ effects on quality, and effects of some subunits have previously been reported [10–13,24–26,40].

Currently, subunits 14+15 [24,41], 13+16, 1, 2* [42] and 5+10 [43] have been shown to have positive effects on quality, while the interactive effects and differences in effects on quality between these subunits were unclear. In this study, the parents of DHs differed in their HMW-GS composition, which consisted of 1 and 2* at *Glu-A1*, 13+16 and 14+15 at *Glu-B1*, and 5+10 and 5'+12 at *Glu-D1*, respectively, and eight subunit compositions in DH lines were distinguished. DHs will be an ideal material for studying favorable subunits effects on the quality of wheat and for detecting subunits interactions underlying specific traits.

### Effects of glutenin subunits on quality

The same subunit may play diverse roles on quality in different HMW-GS compositions. Based on three NILs, Pang et al. [40] demonstrated that the allelic contribution to bread-making quality at the *Glu-B1* locus was in the order of 7+8 > 14+15 > 6+8 > 7 in combination with 5'+12, but when combined with 1 and 5'+12, the ranking was 6+8 > 14+15 > 7. In our study, no significant difference was found in individual subunits at the *Glu-B1* locus, but the effects were varied when combined with other subunits. Tra, ST, and SA (RF) of 14+15 were higher than that of 13+16 for subunit 1 at the *Glu-A1* locus and subunits 5'+12 at the *Glu-D1* locus, whereas in subunit composition SC6 (2*, 13+16, 5+10), 13+16 showed a positive effect on SA, which indicates that the interactive effects between the glutenin loci had a positive contribution to quality traits. For *Glu-A1*, the expression of subunit 1 with subunit pairs 14+15 and 5+10 significantly improved the Zel, while the expression of 2* significantly improved the Rmax (WW) with subunit pairs 14+15 and 5'+12. The present results are in accordance with a previous study, which reported that genotypes with 5+10 have better dough and baking quality, either alone or in combination with other subunits [43].

Materials with favorable subunits are expected to produce better quality, while the theoretical “supper combination” at the *Glu-1* loci may not result in the expected appearance of quality in breeding programs. The optimal combination of HMW-GSs should be underlined in breeding practices in the future. Therefore, understanding the contribution of different HMW-GS combinations to quality will be useful for quality breeding. Our observations suggested that the best performance quality was observed for compositions: 1, 13+16, 5+10 and 1, 14+15, 5+10. This finding provides materials containing favorable alleles at the *Glu-1* loci, which can be used for high-quality gene pyramiding.

Although the influence of background factors is excluded by utilization of a DH population, more comprehensive evaluation of glutenins needs to be considered. Therefore, research on expression levels of *Glu-1* alleles and the structure change and content of GMPs should be conducted in future studies.

### Influence of water regimes on the effects of HMW-GSs on quality

Water deficit affects the expression and accumulation of the grain proteome and causes significant changes in the composition of storage proteins and gluten quality [35]. In general, different HMW-GS components show distinct accumulation patterns under adverse environmental conditions. The expressions of HMW-GS alleles and the proportion of each subunit can be modified by environmental conditions, such as drought and waterlogging [27,35]. A previous study showed that subunits 7+8 were significantly influenced by water, while subunits 14+15, 5+10, and 2+12 were not significantly influenced [44]. Compared with irrigation, the rain-fed regime is beneficial for the accumulation of glutenin in GC8901 (1, 7+8, 5+10), TS23 (1, 7+8, 2+12), and SN139 (N, 14+15, 2+12), as well as the formation of GMPs, and the rate of increase was more obvious in SN139 [44]. Therefore, it is important to reveal the effects of HMW-GS on quality properties when affected by water deficit.

In this study, the Zel, Rmax, and DT of subunits 1, 14+15, and 5+10 were not sensitive to water deficit, whereas the Tra, ST, and SA were significantly affected. This may lead to the accumulation of HMW-GSs and large grain structure of GMP under water deficit, which was in accordance with the results of Zhao et al. [44]. The ST of subunits 5'+12 was significantly different between RF and WW, but showed no differences in combination with other subunits. This indicated that water deficit affects interaction effects on quality traits among subunits at the *Glu-1* loci. The influence of water levels differed among different HMW-GS compositions concerning certain quality traits. For example, the ST and SA of the six subunits were significantly affected by water, but the ST of SC8 (2*, 14+15, 5+10) and SC7 (2*, 14+15, 5'+12) were less affected. Water deficit had a positive effect on the SA of SC4 (1, 14+15, 5+10) and SC3 (1, 14+15, 5'+12), while the other compositions were less affected.

Resulting flour has dough with improved mixing characteristics and water absorption, which can be used to make different types of bread products and noodles [45]. Bread-making needs moderate gluten strength and dough with high extensibility, while noodle-making needs dough with a balance of gluten strength and extensibility to protect the dough from tearing during the manufacturing process [46]. Therefore, it is the balance between elasticity and extensibility that determines the quality of wheat flour for different end-used products [46]. Our observations suggest that Zel and Rmax of the six favorable subunits and eight HMW-GS compositions, as well as DT of subunits 1, 2*, 13+16, 14+15 and 5+10 and the eight HMW-GS compositions, displayed no significant differences between RF and WW. Subunit compositions 1, 13+16, 5+10 and 1, 14+15, 5+10 determined superior bread-making quality. Therefore, appropriate application of water regimes and optimal HMW-GS combination should be utilized in future breeding programs.

## Supporting information

S1 Fig Monthly Rainfall of two years during the growing season. (TIF)

S2 Fig SDS-PAGE(a) of HMW-GSs in DHs and PAGE(b) of PCR products of the DHs using UMN19 marker. a, SDS-PAGE of 8 HMW-GSs compositions, a1- Chinese spring, a2—SC8, a3—SC7, a4—SC2, a5—SC1, a6—SC5, a7—SC4, a8—SC3, a9—SC6, a10—Shiluan02-1; a11—Shaan225, a12—Yannong19. b, DHs were tested with the UMN19 for distinguishing separately 1 and 2*. P1—Jinchun7, P2—L1219.
(TIF)

S1 Table Allele-specific marker for alleles at *Glu-A1* locus in wheat. (XLSX)

S2 Table Correlation coefficients of quality traits between different years and water regimes. (XLSX)

S3 Table Analysis of variance (ANOVA) in quality traits of DHs between two water regimes. (XLSX)

S4 Table Effects of individual subunits on the quality parameters between different water regimes. (XLSX)

S5 Table Bread score of different glutenin subunit composition under two water regimes. (XLSX)
