# Supplementary figures and images for: Effects of HMW-GSs on quality related traits in wheat (Triticum aestivum L.) under different water regimes
Source: PLoS One. 2020 Aug 18;15(8):e0237711. doi: 10.1371/journal.pone.0237711 (PMC7446779; doi:10.1371/journal.pone.0237711)

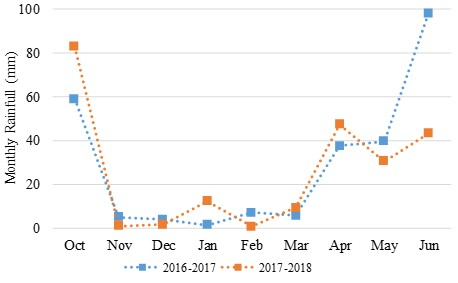

Supplement: S1 Fig — (TIF) [file pone.0237711.s001.tif]

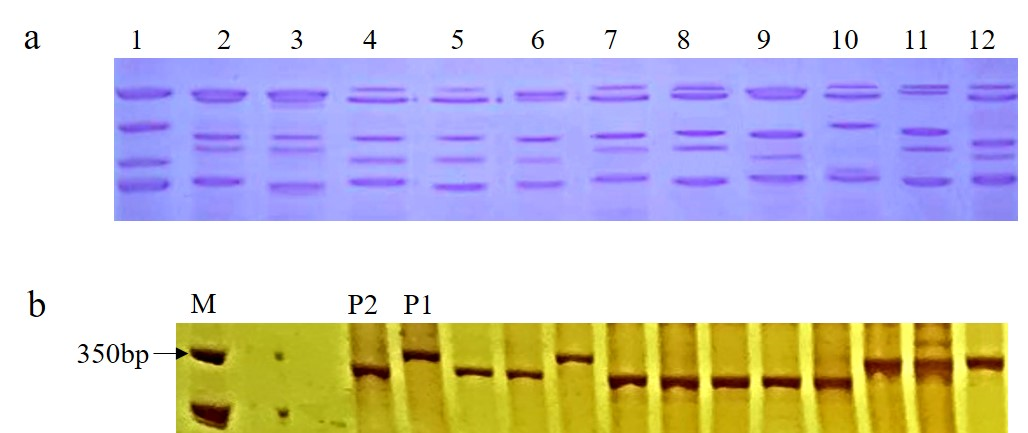

Supplement: S2 Fig — SDS-PAGE(a) of HMW-GSs in DHs and PAGE(b) of PCR products of the DHs using UMN19 marker. a, SDS-PAGE of 8 HMW-GSs compositions, a1- Chinese spring, a2—SC8, a3—SC7, a4—SC2, a5—SC1, a6—SC5, a7—SC4, a8—SC3, a9—SC6, a10—Shiluan02-1; a11—Shaan225, a12—Yannong19. b, DHs were tested with the UMN19 for distinguishing separately 1 and 2*. P1—Jinchun7, P2—L1219. (TIF) [file pone.0237711.s002.tif]
